# Molecular Pathogenesis of Cholangiocarcinoma

**DOI:** 10.1155/2012/630543

**Published:** 2011-07-21

**Authors:** G. Fava, I. Lorenzini

**Affiliations:** Division of Gastroenterology, Azienda Ospedaliero-Universitaria “Ospedali Riuniti” Via Conca 72, 60020 Ancona, Italy

## Abstract

Epidemiological data from the last years show an increasing trend of incidence and mortality of cholangiocarcinoma (CC) worldwide. Many pathophysiologic aspects of this neoplasia are still unknown and need to be fully discovered. However, several progresses were recently made in order to establish the molecular mechanisms involved in the transformation and growth of malignant cholangiocytes. The principal concept that at least seems to be established is that cholangiocarcinogenesis is a multistep cellular process evolving from a normal condition of the epithelial biliary cells through a chronic inflammation status ending with malignant transformation. The bad prognosis related to CC justifies why a better identification of the molecular mechanisms involved in the growth and progression of this cancer is required for the development of effective preventive measures and valid treatment regimens. This Paper describes the scientific progresses made in the last years in defining the molecular pathways implicated in the generation of this devastating disease.

## 1. Introduction

Cholangiocarcinoma is originated by a malignant transformation of cholangiocytes, the epithelial cells lining the biliary ducts [1]. Since biliary cancers may arise from every portion of the biliary tree, they are anatomically classified as intrahepatic or extrahepatic [1]. Epidemiological data show that intrahepatic cholangiocarcinoma is increasing in incidence, prevalence, and mortality worldwide [2, 3]. In particular, in the past three decades, a progressive increase of mortality for intrahepatic CC has been reported, while mortality for extrahepatic CC is stable or slightly decreasing [3]. 

The poor prognosis of this cancer is also explained from the fact that no useful tools for early diagnosis for this neoplasia are still available. Because of, the lack of specific symptoms coupled with high invasiveness and frequent involvement of critical anatomical organs [1, 4], the patient typically presents with an unresectable disease at the diagnostic approach. This aspect justifies why a surgical curative treatment is often impossible. Besides surgery, the other types of treatments for CC are chemotherapy and radiotherapy [1]. However, CC cells do not respond or weakly respond to these approaches, which thus have often a palliative role. Recent therapeutic options include brachytherapy and photodynamic therapy (PDT), with promising results [1, 4].

CC develops from the accumulation of genetic and epigenetic alterations in regulatory genes in cholangiocytes that lead to the activation of oncogenes and the dysregulation of tumor suppressor genes (TSGs) [5–8]. The principal characteristics of malignant cholangiocytes can be summarized in (i) uncontrolled growth, (ii) high capacity of tissue invasiveness, and (iii) capacity to metastasize [5, 8]. In this paper, we have described in detail the principal molecular mechanisms involved in every passage of the multistep process of cholangiocarcinogenesis.

## 2. Molecular Mechanisms of Cholangiocarcinogenesis

**Figure 1:**
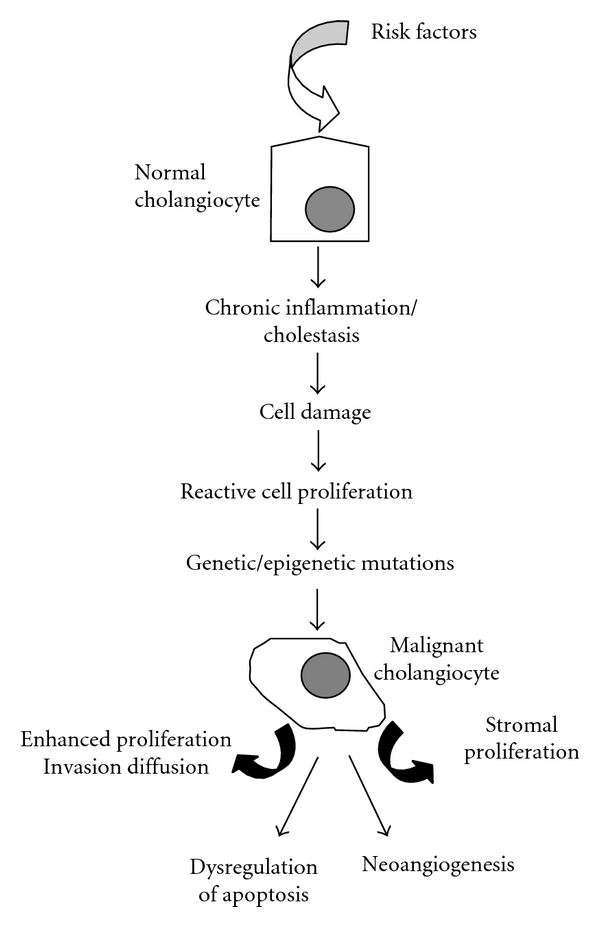
Proposed mechanisms leading to transformation of normal biliary cells into malignant cholangiocytes. Cholangiocarcinoma cells express altered molecular mechanisms, which enhance cell proliferation, decrease apoptosis, and increase the capacity of tissue invasion, stromal proliferation, and angiogenesis.

The molecular mechanisms involved in the development of CC are incompletely defined in detail, although in the last years, several studies have contributed to codify them, at least in part [5]. With the term “cholangiocarcinogenesis” are named all the complex mechanisms that lead to the malignant transformation of cholangiocytes. These mechanisms can be simply described as a multistep process (Figure 1). CC usually develops in an environment of chronic inflammation of bile ducts with consequent cholangiocyte damage associated with the obstruction of bile flow [1, 5]. This tumor, especially when originating in the perihilar bile ducts, can develop in normal liver [5, 9]. 

Primary sclerosing cholangitis (PSC) is the most recognized risk factor for CC development [9]. Other risk factors for this cancer are specific parasites of endemic regions of Asia such as *Opisthorchis viverrini*, *Clonorchis sinensis*, and *Schistosoma Japonica *and bacteria such as *Salmonella typhi* [9, 10]. Hepatolithiasis, Caroli's disease, congenital choledochal cysts, bilioenteric surgical drainage and anomalous pancreaticobiliary junction, age greater than 65 years, bile duct adenoma, papillomatosis, liver cirrhosis, smoking, diabetes mellitus, thorotrast, dioxin and vinyl chloride intoxication, and HIV and HCV infections [9, 10] are also risk conditions for CC. However, the role of most of these conditions as predisposing factors for biliary cancer is still debated. Independently from the presence of one of the mentioned factors, the malignant transformation of cholangiocyte arises in a background of chronic inflammation. The high amount of cytokines and factors secreted during chronic inflammatory processes triggers and maintains the process of cholangiocarcinogenesis [5, 8] (Figure 1). Molecules participating in chronic inflammation promote neoplastic process by damaging protooncogenes, DNA mismatch repair genes/proteins, and tumor suppressor genes involved in cell growth, apoptosis, invasiveness, and neoangiogenesis. The final result is the uncontrolled cell proliferation and invasion (Figure 1).

Such as in many other cancer types, K-ras, p53, p14ARF, p16INK4a, and *β*-catenin genes can be mutated during the development of CC [6].

Two other genes recently described as implicated in the development of CC are *NKG2D* and *AID*. The natural killer group 2, member D cell receptor, also known as NKG2D, is expressed by NK cells and T-lymphocytes and plays a critical role in tumor surveillance by cell-mediated cytotoxicity [11]. Melum et al. recently showed that two single nucleotide polymorphisms (SNPs) of the NKG2D gene were associated with an increased risk of CC in PSC-affected patients [12]. Contrarily, homozygous condition for the no-risk alleles is related with a low risk of CCs [12].

Activation-induced cytidine deaminase (AID) is a member of the DNA/RNA-editing enzyme family. Recently, it was shown that AID production was significantly increased in human biopsies of PSC and CC-affected patients compared with normal liver parenchyma [13]. Aberrant expression of AID in biliary cells resulted in the generation of somatic mutations in tumor-related genes such as *p53*, *c-myc *and the promoter region of the *INK4A/p16 *sequences [13]. The aberrant expression of AID gene induced by proinflammatory cytokines strengthens the link between chronic inflammation of the biliary tract and CC development [13].

## 3. Molecular Pathways Implicated in Cholangiocarcinogenesis

### 3.1. IL-6

IL-6 has an important role in the pathogenesis and growth of CC [14]. The mitogenic effect of IL-6 is suggested from the fact that the concentration of this molecule is increased during chronic inflammation of the biliary tract, a condition predisposing CC development [15]. IL-6 acts by both an autocrine and a paracrine manner stimulating several intracellular pathways involved in survival and growth of malignant cholangiocytes [16]. Among them, p44/p42 and p38 MAPKs have been largely studied [17]. Tadlok et al. showed that activation of p38 MAPK by IL-6 decreases expression of p21(WAF1/CIP1), a cell cycle controller protein, and mediates growth independent of anchorage signals, whereas activation of p44/p42 MAPK mediates an anchorage signal-dependent growth pathway [17]. IL-6 also influences the apoptotic process of malignant cholangiocytes. Several studies showed that IL-6 upregulates myeloid cell leukemia-1 or Mcl-1, a potent key antiapoptotic Bcl-2 family member protein. It has been recently shown that this effect of IL-6 is mediated by increased activation of STAT-3 (that is constitutively activated in malignant cholangiocytes), which regulates Mcl-1 transcription thus increasing resistance to apoptosis [18]. In addition, Mcl-1 increases cancer cell resistance to tumor-necrosis-factor-related apoptosis-inducing ligand (TRAIL) [19] and, therefore, this molecule appears to have a fundamental role in CC development [20]. Conversely, inhibition of IL-6-induced iperexpression of Mcl-1 restores sensitivity to TRAIL [21].

### 3.2. JAK/STAT

The Janus kinase/signal transducer and activator of transcription (JAK/STAT) pathway is one of the key signaling mechanisms in CC cells, mediating their resistance to apoptosis [20]. In a recent study, Blechacz et al. showed that the multikinase inhibitor sorafenib may also block JAK/STAT signaling with consequent inhibition of CC growth [22]. The authors demonstrated that sorafenib induces STAT3 dephosphorylation by stimulating phosphatase SHP2 activity, sensitizes CC cells to TRAIL-mediated apoptosis, and is therapeutic in a syngeneic rat, orthotopic CC model that mimics human disease [22].

### 3.3. TGF*β*

TGF**β** is a cytokine implicated in several cell functions such as growth, differentiation, migration, apoptosis, adhesion, survival, and immunity. Several cell types of the liver secrete this cytokine [21]. Cholangiocytes, for example, express TGF*β* in course of cholestasis but not in normal conditions [23]. It has been shown that TGF*β* inhibits proliferation of human CC cells through modulation of the p21 cyclin dependent kinase inhibitor [24]. However, the mutations of TGF*β* receptor and the alterations of intracellular signaling mediators (e.g., Smad4) together with the intracellular overexpression of cyclin D1 [25, 26] in CC cells induce a resistance to the inhibitory effect of TGF*β* cells [27]. In addition, the lack of TGF*β* signaling also stimulates the deposition of fibrotic tissue, abundantly expressed by biliary malignancies [27].

### 3.4. Smad4

DCP4/Smad4 is a tumor suppressor gene and also a downstream of TGF*β* signaling [28]. Recently, it has been shown that Smad4 interacts with PTEN, another tumor suppressor gene, in order to regulate cellular cycle and escape the process of cholangiocarcinogenesis [29]. To strength this data, the blockage of these tumor suppressor genes favors the development of CC [29, 30]. It was also demonstrated that pTNM stage of intrahepatic CC appears to be correlated with the degree of Smad4 loss [30].

### 3.5. ErbB-2

The protooncogene-encoded receptor tyrosine kinase ErbB-2 is overexpressed in malignant cholangiocytes and plays an important role in the development and progression of biliary malignancies [31–33]. ErbB-2 acts in two different manners: first of all stimulates the proliferation of CC cells, then ErbB-2 stimulates the production of COX-2, which interacts with a subunit of the IL-6 receptor forming a complex [34]. This effect suggests a close link between IL-6 and ErbB-2 signaling [1, 34]. The mitogenic effect of ErbB-2 is also suggested from the fact that when normal cholangiocytes are transfected with the neu (the rat homologue of ErbB-2) oncogene, they undergo a malignant transformation that resembles the molecular aspects of human CC [35].

### 3.6. COX-2

The enzyme cyclooxygenase (COX) is responsible of the generation of prostaglandins, expressed in the course of the process of inflammation. COX exists in two specific isoforms: COX-1, normally expressed in many cell types and regulating the homeostatic functions of prostaglandins, and COX-2, the inducible isoform, which can be stimulated by a variety of molecules, such as cytokines and lipopolysaccharides [36]. The expression of COX-2 is major during inflammation, condition predisposing the development of CC [36]. Moreover, in rat CC cells, overexpression of COX-2 stimulates cell growth [37] while antisense depletion of COX-2 inhibits cell proliferation [37]. Recent studies showed that COX-2 is activated in human CC cells *in vitro* [38] by oxysterols, derivatives from cholesterol, which are present in bile of patients in course of cholestasis and inflammatory processes of the biliary tract [39]. The mitogenic effect of COX-2 towards CC cells justifies why the inhibition of COX-2-mediated pathway could represent a strategy to prevent CC development and growth. At this regard, recent data demonstrated that selective COX-2 inhibitors (e.g., celecoxib) reduce proliferation of CC cells by stimulating apoptosis [31, 37, 40, 41]. These studies also showed that the inhibitory effect towards CC cell growth by celecoxib was accompanied by an inhibition of PDK1 and PTEN, with a consequent reduction of Akt phosphorylation [42]. Moreover, celecoxib inhibits CC cells proliferation through activation of cyclin-dependent kinase inhibitors p21^waf1/cip1^ and p27^kip1^, with consequent cell cycle arrest at G1/S phase [43]. Sirica et al. recently demonstrated a link between the increase of COX-2 expression and CC development since the amount of this cytokine is high in cholangiocytes obtained from livers affected by PSC [31], a well known risk factor for CC [9]. These promising *in vitro* data, however, do not correspond to a good outcome in clinical practice since not all COX-2 inhibitors were shown to have a benefit in reducing CC cell growth [37] and also because the use of high doses of COX-2 inhibitors could cause serious side effects [37].

### 3.7. NO

Inducible nitric oxide (NO) synthase (iNOS) is an enzyme highly expressed during inflammatory and malignant processes of the biliary tract [44]. The activation of iNOS in course of inflammation determines an increase of intracellular NO, which triggers the process of cholangiocarcinogenesis by different ways: (1) inhibits DNA repair system thus allowing an accumulation of DNA damage and mutations [8, 45]; (2) stimulates COX-2 expression [44].

The carcinogenic effect of NO is at least in part due to Notch-1 signaling [46]. The role played by NO and Notch-1 in the development of biliary malignancies, as well as pancreatic cancer, is suggested by the evidence that Notch-1 is hyperexpressed both in cholangiocytes of PSC-affects patients and in CC cells where it colocalizes with iNOS [46]. A recent study by Ishimura et al. showed that iNOS is able to stimulate COX-2 expression through NO generation. In particular, iNOS enhances COX-2 expression through activation of p38 MAPK and JNK1/2 [44]. The link between these two proteins explains their important role in the development and growth of CC [44].

### 3.8. Apoptosis

The process of apoptosis is fundamental to maintain the homeostasis of the biliary epithelium because it permits to remove the cells deeply damaged and with no reversible genomic mutations [47, 48]. After this premise, it is clear that a defect of apoptotic process favorites the survival of mutated cholangiocytes, which could go through a series of other mutations ending with the malignant transformation of the cell [47, 48].

Bcl-2 is a superfamily of antiapoptotic proteins. Bcl-2, which represents the prototype of this family [49] and is expressed in CC cells in a high amount. In these malignant cells, which possess a higher apoptotic threshold with respect to normal cholangiocytes [49], Bcl-2 exerts its antiapoptotic activity by reducing caspase 3 activation by preventing cytochrome-c release from the mitochondria [49]. 

Several other factors are implicated in the dysregulation of cholangiocyte apoptosis [8]. Among them, NO inhibits apoptosis of biliary epithelial cells. At this regard, the transfection of CC cells with nitric-oxide-synthase- (NOS-) cDNA induces a resistance to etoposide-induced apoptosis, an event that happens by caspase 9 nitrosylation [50].

Furthermore, Notch-1 and COX-2 reduce TRAIL-mediated apoptosis [21] and high levels of COX-2 inhibit Fas-induced apoptosis in CC cells [21]. To strengthened these data, a recent study demonstrated that celecoxib, a selective COX-2 inhibitor, induces cell death by apoptosis by the inhibition of the PI3-kinase signaling [37, 41]. 

The cytokine tumor-necrosis-factor-related apoptosis-inducing ligand (TRAIL) selectively stimulates apoptosis only in malignant cells without having any toxicity in normal tissues [51, 52]. CC cells resist TRAIL-induced apoptosis because they express high levels of myeloid cell leukemia protein-1 (Mcl-1) [19]. Thus, when specific small-interfering mRNA or stable transfection with Mcl-1 small hairpin RNA block Mcl-1 expression, CC cells become sensitive to TRAIL-induced apoptosis [19, 53]. The expression of Mcl-1 is also stimulated by bile acids, abundant in the course of cholestasis. Among them, deoxycholic acid, for example, increases Mcl-1 expression by blocking protein degradation through activation of an EGFR/Raf-1 pathway [54]. Indeed, Raf-1 inhibitors block the increase of Mcl-1, rendering the cells much more sensitive to Fas-induced apoptosis [55]. All these data suggest that TRAIL could be a target for novel drugs for the management of biliary tumors [56].

### 3.9. VEGF

Biliary tumors proliferate surrounded by a rich vascular network, which provides an adequate support of oxygen and metabolites to malignant cholangiocytes in order to enhance tumor development and growth [1, 57]. The proliferation of blood vessels is favored by high levels of vascular endothelial growth factor (VEGF) [57, 58]. This protein is stimulated by TGF-*β* and *β*-catenin [59] and is expressed by the surrounding mesenchymal cells and, even if in a lesser extent, by the malignant cells themselves. This evidence suggests the existence of an autocrine/paracrine mechanism for the VEGF production by malignant cholangiocytes [60].

### 3.10. Estrogens

It is well known the mitogenic effect of estrogens for CC cells. At this regard, 17-*β* estradiol stimulates human CC cells growth [61] while tamoxifen, an estrogen antagonist, decreases the proliferation of these cells *in vitro* and *in vivo* [61] by stimulating apoptosis through the Fas/APO-1 (CD95) signaling pathway via a calmodulin-dependent mechanism [62]. Alvaro et al. demonstrated that human intrahepatic CC cells express receptors for both estrogens and insulin-like growth factor 1 (IGF-1), which cooperate in the modulation of enhancing cell growth and reducing apoptosis [63]. Furthermore, HuH-28 human intrahepatic CC cell line expresses VEGF-A, VEGF-C, and related receptors, which are enhanced by stimulation with estrogens [64], and the stimulatory effect of 17beta-estradiol is blocked by estrogen receptor or insulin-like growth factor-1 receptor antagonists [64]. These data demonstrate that estrogens stimulate the proliferation of human CC by inducing the expression and secretion of vascular endothelial growth factor [64]. The result of these studies could be applied to the management of biliary malignancies in clinical practice. In fact, measuring IGF-1 levels in bile could help distinguish extrahepatic CC from pancreatic cancer or benign biliary stenosis and blocking estrogen, IGF-1, and VEGF receptors could be crucial to arrest CC cell proliferation [65].

### 3.11. Neuropeptides and Hormones

In the last years, many studies described as a multitude of hormones and neuropeptides are able to interact and regulate CC cells growth and invasion. Among them, gastrin, endothelin, serotonin, secretin, histamine, the *α*-2-adrenoreceptor UK14, 304, NPY, GABA, leptin, and opioid receptor modulators regulate the proliferation and apoptosis of CC cells [66–75]. All these novel data could contribute to clarify the complex mechanisms governing the process of cholangiocarcinogenesis.

## 4. Conclusion

The increasing worldwide incidence of CC together with the lack of its effective therapeutic tools explains the growing general interest for the study of this cancer type. The bad prognosis of people affected by CC is given by the fact that this cancer is often diagnosed when already at an advanced stage. Unfortunately, at this point, only palliative approaches are possible. With these premises, in the recent years, many researchers have focused their studies on the investigation of the molecular mechanisms involved in the development and growth of CC. Several works demonstrated that the conditions of cholestasis and chronic inflammation induce a local release of a network of mitogenic factors that induce genomic damages thus triggering the malignant transformation of cholangiocytes. The complete codification of molecular pathways involved in the pathogenesis of CC is mandatory to discover novel tools for an early diagnosis and an efficacious specific therapy.
